# Bit-Level Automotive Controller Area Network Message Reverse Framework Based on Linear Regression

**DOI:** 10.3390/s22030981

**Published:** 2022-01-27

**Authors:** Zixiang Bi, Guoai Xu, Guosheng Xu, Chenyu Wang, Sutao Zhang

**Affiliations:** School of Cyberspace Security, Beijing University of Posts and Telecommunications, Beijing 100876, China; bzx@bupt.edu.cn (Z.B.); guoshengxu@bupt.edu.cn (G.X.); wangchenyu@bupt.edu.cn (C.W.); lunter-zst@bupt.edu.cn (S.Z.)

**Keywords:** Controller Area Network, electronic control units, database CAN, reverse, multiple linear regression, bit-level, vehicle behavior

## Abstract

Modern intelligent and networked vehicles are increasingly equipped with electronic control units (ECUs) with increased computing power. These electronic devices form an in-vehicle network via the Controller Area Network (CAN) bus, the de facto standard for modern vehicles. Although many ECUs provide convenience to drivers and passengers, they also increase the potential for cyber security threats in motor vehicles. Numerous attacks on vehicles have been reported, and the commonality among these attacks is that they inject malicious messages into the CAN network. To close the security holes of CAN, original equipment manufacturers (OEMs) keep the Database CAN (DBC) file describing the content of CAN messages, confidential. This policy is ineffective against cyberattacks but limits in-depth investigation of CAN messages and hinders the development of in-vehicle intrusion detection systems (IDS) and CAN fuzz testing. Current research reverses CAN messages through tokenization, machine learning, and diagnostic information matching to obtain details of CAN messages. However, the results of these algorithms yield only a fraction of the information specified in the DBC file regarding CAN messages, such as field boundaries and message IDs associated with specific functions. In this study, we propose multiple linear regression-based frameworks for bit-level inversion of CAN messages that can approximate the inversion of DBC files. The framework builds a multiple linear regression model for vehicle behavior and CAN traffic, filters the candidate messages based on the decision coefficients, and finally locates the bits describing the vehicle behavior to obtain the data length and alignment based on the model parameters. Moreover, this work shows that the system has high reversion accuracy and outperforms existing systems in boundary delineation and filtering relevant messages in actual vehicles.

## 1. Introduction

The increasingly diverse features in today’s vehicles offer drivers and passengers a more relaxed driving experience and greater convenience. Vehicle connectivity provides real-time information and a variety of entertainment options. In addition, vehicle support features such as advanced driver assistance systems (ADAS), reduce driving stress and make driving safer. These capabilities have multiplied due to the increasing number of electronic control units (ECUs) and higher computing power. Current vehicles are equipped with up to 150 ECUs [1], that need to communicate in a unified network that requires the vehicles to provide sophisticated real-time performance, sufficient data transmission volume, and adequate reliability. Control Area Network (CAN), a technology that meets these requirements, became the international standard for intra-vehicle network communication in 1993 [2]. However, since CAN uses broadcast communication and lacks security mechanisms such as encryption and authentication, it increases the probability that the vehicle will be attacked [3,4,5,6].

Many examples of attacks on vehicles have confirmed that it is possible to attack the vehicle and perform negative control [7,8,9]. The most typical attack case is the attack by Miller et al. on a Jeep Cherokee that was driving on the highway and used a CAN bus-connected entertainment system and ECU firmware, that resulted in acceleration and brake failures [10]. More recently, Keen Labs in China exploited vulnerabilities in Tesla’s assisted driving system to drive the vehicle into the reverse lane and even remotely control the vehicle’s steering with a gamepad [11]. Regardless of the type of vulnerability, the common denominator of the attack is the need to inject information into the CAN bus to cause the vehicle to behave dangerously [12]. To prevent the CAN bus from being infiltrated with targeted attacks, original equipment manufacturers (OEMs) privatize the database CAN (DBC) file. The DBC file defines the structure, content, and meaning of each message in the CAN network [13,14]. Even the DBC file is different for different models of the same brand. It is very time-consuming for an attacker to work reverse before implementing CAN bus attacks. For security researchers, private DBC files are a massive obstacle to CAN security research. The most affected area is the automotive intrusion detection system (IDS), a crucial research element in automotive security. CAN intrusion detection systems have been proposed to detect anomalies by analyzing CAN traffic [15,16,17,18,19,20,21,22,23], but these studies are based on message transmission characteristics that are practically irrelevant to the behavior and status of the vehicle. Therefore, the existing IDSs for the CAN are not very powerful. Another hindered study is the fuzzy test on the CAN bus, which is often used to automatically test and discover unknown vulnerabilities in ECUs [24,25,26,27,28]. Since the DBC files are hidden, which causes the fuzzy test intelligence to construct data blindly, brute force and random data make the test inefficient. In addition, the lack of DBC files with detailed descriptions of CAN messages hinders automotive aftermarket development. Without effective access to vehicle status, automotive driver assistance systems and status display tools become meaningless.

The detailed specification of CAN messages is crucial for CAN network intrusion detection, fuzz testing, and automotive aftermarket products. To obtain the CAN message description in the DBC document, the security research field has proposed CAN bus reversion methods such as CAN message tokenization algorithm, machine learning-based inversion method, and onboard diagnostics II (OBD-II) diagnostic information matching. The earliest CAN message tokenization algorithm was the FBCA algorithm proposed by Markovitz et al. in 2017 [29], followed by the READ algorithm proposed by Marchetii and Stabili in 2018 [30]. The automatic CAN message translator LibreCAN was proposed by Pesé et al. in 2019 [31]. Recently, the ReCAN [32] dataset was published by Zago et al. in 2020 using a similar approach to READ. However, they are limited to classifying and subdividing data changes, such as constants, multiple values, counters, sensors. These cannot obtain specific information, such as the meaning and alignment of each tagged data. It is of minimal help for IDS research and aftermarket. The most typical of the machine learning-based CAN message reversal methods are Jaynes et al. proposed a method for efficient identification of sending ECUs, which identifies CAN frame by analyzing a similarity construction model describing uniform vehicle state information [33]. A data-driven CAN bus reversion method proposed by Buscemi et al. used already available open-source DBC files to train a machine learning model to identify unknown CAN message contents [34], a scheme similar to the unsupervised machine learning-based scheme proposed by Ezeobi et al. [35]. The accuracy of this type of solution depends entirely on the coverage of the training set. Since each vehicle is configured with a unique DBC file, it is almost impossible for the training set of such algorithms to cover all vehicle models. These approaches have been validated only on simulated data and are practically infeasible. Methods based on matching OBD-II diagnostic information describe the vehicle status in CAN information by comparing and matching OBD-II responses. Song and Kim et al. first proposed to create windows before and after the OBD-II response information to find candidate information that exactly duplicates the response data and repeat it several times to determine the information describing the response [36]. Blaauwendraad proposed a matching method using correlation coefficients based on Song’s method [37]. While these methods can yield some inversion results, they can only identify specific vehicle behavior in CAN messages. The insufficient number of supported vehicle behaviors for per-vehicle diagnostics limits the application of this scheme. Additionally, the CANHUNTER [38] proposed by Wen et al. in 2020 reverses the CAN message by disassembling the control APP that interacts with the car. Although this is a novel idea, this method can only obtain what is specified in the APP, and the scheme will be completely invalid once the APP commands are escaped at the server-side. In addition, since such APPs are only valid for the specified car model, this scheme also receives the limitation of the car model. In summary, existing CAN message reversal techniques are limited in their implementation by the number of available DBC files and vehicle models, and their results are unsatisfactory. Solutions that are not limited by vehicle models and can achieve close to the DBC file reversal results are urgently needed.

The CAN frame data tags alone do not reveal any valuable information, and one needs to have DBC files to decode them. However, the DBC files are hidden and usually different for each model. Reverse engineering solutions for CAN information that are not constrained by the vehicle model and can access critical information in the DBC files are urgently needed. To achieve CAN message reversal close to the DBC file, this study innovatively proposes a multiple linear regression model after an in-depth analysis of the way the DBC file specifies the vehicle behavior. The model is built using each bit of the CAN message data field as the independent variable and the vehicle behavior data as the dependent variable. As the input of our framework additionally includes sensor data, our framework needs to be very useful. First, the framework uses the $R^{2}$ of the model to filter the candidate messages related to vehicle behavior, which has an excellent filtering result on related messages compared to existing schemes. In addition, the framework outperforms existing systems in terms of data boundary delineation by locating the bits describing the vehicle behavior and obtaining the details of field functions, starting bits, field lengths, and alignment formats in the DBC file based on the β value of each model. Finally, since commercially available vehicles must be configured with a standard CAN data interface and the vehicle behavior can be captured by commonly used sensors, the inverse framework proposed in this study is independent of the vehicle model and brand.

The structure of this study is as follows. Section 2 introduces the CAN bus, DBC file, multiple linear regression models preliminary introductions and describes the feasibility of the study’s ideas. Section 3 describes the design and implementation ideas of the framework. Section 4 evaluates the performance of the CAN reverse framework in actual vehicles, the reverse accuracy, the time required, the advantages over existing solutions, and the applicability of the framework. Section 5 concludes the study.

## 2. Background and Feasibility

### 2.1. CAN Bus Overview

The CAN bus is a serial communication bus originally developed by Bosch [39]. Later the international standards organization (ISO) issued the international standard ISO11898 for CAN in 1993 [40]. CANs have become one of the most widely used fieldbuses globally due to their high transmission rate and high real-time characteristics.

**Figure 1:**
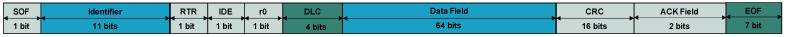
Standard CAN message frame.

The standard format of a CAN message is shown in Figure 1. It begins with the start of frame (SOF), followed by an 11-bit identifier (ID) and a remote transmission request (RTR). The ID defines the meaning and type of the message and is also used to filter irrelevant messages when the node receives the messages. The ID is also used for arbitration when multiple nodes send data simultaneously; the smaller the ID is, the higher the priority is. RTR is used to distinguish the type of message. A six-bit control field follows this: identifier extension (IDE) and r0 specify the length of the frame, and the data length code (DLC) specifies the number of bytes in the data field. The data field is the core of the CAN message and is 64 bits long. It contains the vehicle control commands, the status data, and any other data to be transmitted (e.g., counters, checksum values, etc.). This is followed by the Circular Check Code (CRC), the Acknowledgement Field (ACK), and the end of frame (EOF), respectively.

For CAN message reversal work, the main targets of the reversal are the identifier (ID) and the data fields. When reversing CAN messages, the relevant message ID is usually locked first, and then the data fields are analyzed to obtain specific bit fields that characterize the vehicle behavior.

### 2.2. DBC File

The form and content of each type of CAN message are defined in the DBC file, so each OEM keeps it private to avoid leakage from the data source and prevent negative control and modification of the car. However, all CAN messages must be fully translated using the DBC file as a table, making sense for CAN reverse work. The contents defined in the DBC file are listed in Table 1. The Name, ID, Cycle Time, and Length describe the entire message. The Function specifies one or more vehicle behaviors in the message data fields. Byte Order, Start Byte, Start Bit, Bit Length, Units, Precision, and Offset specify how the message describes the specific behavior. Typically, the data fields of a message contain multiple functions.

**Figure 2:**
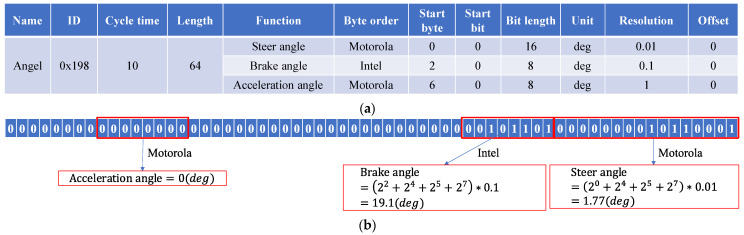
Correspondence diagram between DBC file and CAN messages: (**a**) 0x198 Message definition in DBC; (**b**) Message data decoded according to DBC.

The message with ID 0x198 is used to explain the correspondence between the DBC file and the CAN message content. As shown in Figure 2a, the DBC file defines the name of the message as angle, the message sending period is 10 ms, the message length is 64 bits, and it contains 3 vehicle behaviors: steering angle, brake pedal angle, and gas pedal angle. The steering angle is arranged in Motorola (LSB) form from bit 0 to bit 15 with a resolution of 0.01. Similarly, the gas pedal and brake pedal angles are arranged in bits 16–23 and 48–55 of the data field, respectively. The alignment is Intel (MSB) and Motorola. When capturing any message with ID 0x198, its data can be decoded according to the provisions of the DBC file. According to the definition of DBC, the message shown in Figure 2b describes the angle information of the vehicle at this moment, where the brake pedal angle is $\left(2^{2}+2^{4}+2^{5}+2^{7}\right)\times 0.1=19.1{}^{\circ}$, the steering angle is $\left(2^{0}+2^{4}+2^{5}+2^{7}\right)\times 0.01=1.77{}^{\circ}$, and the throttle angle is 0.

In summary, the DBC file is vital to study the CAN messages in-depth, which makes the DBC file a realistic target for reverse work.

### 2.3. Linear Regression Preliminary

In statistics, the multiple linear regression model describes the linear relationship [41,42] between the scalar dependent variable y and several explanatory variables defined as $X=\left(x_{1},x_{2},\ldots ,x_{k}\right)$ and the model function is shown in Equation (1), where $\beta =\left(\beta_{0},\ldots ,\beta_{k}\right)$ is an unknown model parameter that can be estimated by giving sample set of y and X. The ordinary least squares method is the most commonly used method for parameter estimation. For a given sample set $y_{e}\;$(see Equation (2)) and $X_{e}$ (see Equation (3)), the ordinary least squares method first creates a new matrix Ω, as shown in Equation (4).

(1) $$y=\beta_{0}+\beta_{1}x_{1}+\beta_{2}x_{2}+\ldots +\beta_{k}x_{k}$$

(2) $$y_{e}=\left(\begin{array}{c} y_{1}\\ y_{2}\\ \vdots \\ y_{m} \end{array}\right)$$

(3) $$X_{e}=\left(\begin{array}{ccc} x_{11} & \ldots & x_{1k}\\ x_{21} & \ldots & x_{2k}\\ \vdots & \ddots & \vdots \\ x_{m1} & \ldots & x_{mk} \end{array}\right)$$

(4) $$\Omega =\left(\begin{array}{cccc} 1 & x_{11} & \ldots & x_{1k}\\ 1 & x_{21} & \ldots & x_{2k}\\ \vdots & \vdots & \ddots & \vdots \\ 1 & x_{m1} & \ldots & x_{mk} \end{array}\right)$$

The estimation $\hat{\beta }$ can be obtained from Equation (5), where $\Omega^{T}$ is the transpose of Ω. The determination coefficient $R^{2}$ indicates how well the samples fit the linear model created with $\hat{\beta }$ and is calculated by Equation (6), where ${\hat{y}}_{i}={\hat{\beta }}_{0}+{\hat{\beta }}_{1}x_{i1}+\ldots +{\hat{\beta }}_{i}x_{ik}$ is the $y_{i}$ estimated with the linear model and ${\bar{y}}_{i}$ is the mean of $y_{e}$. The value of $R^{2}$ is in the range $\left[0,1\right]$, and 1.0 is the best fit.

(5) $$\hat{\beta }={\left(\Omega^{T}\Omega \right)}^{-1}\Omega^{T}y_{e}$$

(6) $$R^{2}=1-\frac{\sum_{i=1}^{m}{\left(y_{i}-{\hat{y}}_{i}\right)}^{2}}{\sum_{i=1}^{m}{\left(y_{i}-\bar{y_{i}}\right)}^{2}}$$

### 2.4. Feasibility

Based on the way the CAN messages are defined in the DBC file and the characteristics of the multiple linear regression model, this section presents the feasibility of a bit-level inverse CAN message.

**Figure 3:**
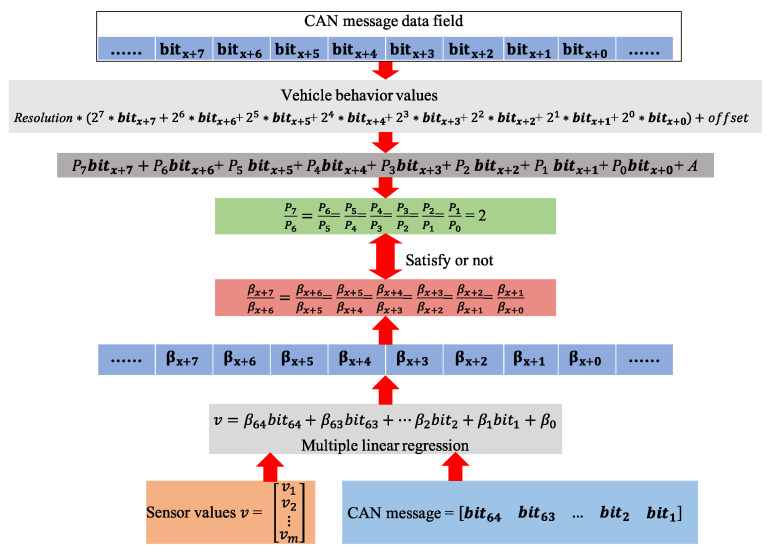
Reverse feasibility based on linear regression.

According to the definition of the DBC file, the vehicle behavior in the CAN message is expressed as a binary serial number of bits, and there is also a resolution and an offset between the actual vehicle behavior data and this value. As shown in Figure 3, the relationship between the actual vehicle behavior and the corresponding bits in the CAN message is linear, and the adjacent linear coefficients satisfy the two-fold relationship. A multiple linear regression model of sensor data and each bit in the CAN message can be constructed when sensors are used to obtain vehicle behavior data. If the adjacent regression coefficients β satisfy the doubling relationship, the consecutive bits corresponding to the coefficients describe the vehicle behavior. In addition, the length, boundary, and alignment of the data can be determined based on the β that satisfies the condition.

## 3. Framework Design

According to the previous description, the DBC file defines the detailed content and form of each message, which is critical for both the research and aftermarket communities. For the scientific field, obtaining the specific meaning of CAN messages facilitates the construction of better Intrusion Detection Prevention Systems (IDPS), instead of just finding anomalies based on data variation patterns. In addition, fuzzy testing can also improve efficiency by performing more targeted data injections based on the content of CAN messages. For aftermarket manufacturers, DBC files can help produce more driver assistance products, such as head-up displays and driver assistance devices. However, for confidentiality and security reasons, OEMs keep DBC files private. In addition, most of the existing CAN message reversal solutions are focused on sorting and ID filtering of data fields. The current CAN message reversal results are limited, obtaining the tags of the data types, data boundaries, and the message IDs associated with some car behaviors.

**Figure 4:**
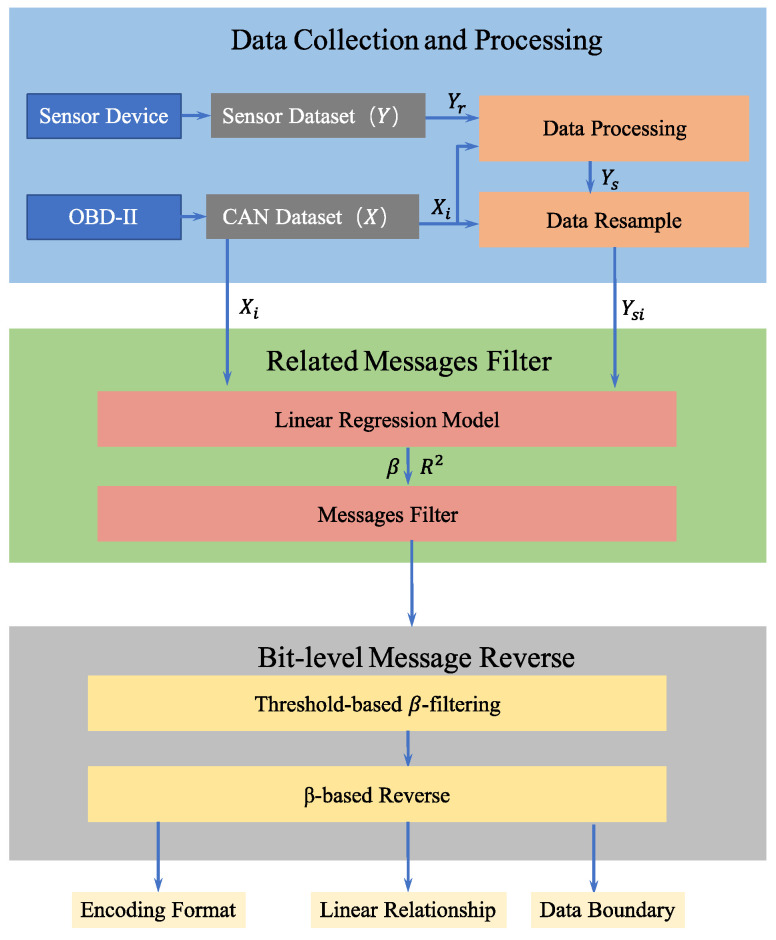
Overview of the framework.

In this study, a bit-level automotive CAN message reverse framework is proposed by building a multiple linear regression model for CAN message data fields and actual physical measurements of the vehicle. Based on the optimal model parameters, the messages related to vehicle behavior are filtered. The data content, data boundary, encoding format, and linear relationship of CAN messages are extracted to maximize the recovery of the DBC file. Figure 4 provides an overview of the framework in three phases: data collection and processing, related message filtering, and bit-level message reverse. The variables used in each phase are defined below.

- X: the raw CAN dataset of the vehicle obtained from the OBD-II interface, containing the entire behavioral trajectory of the vehicle.
- Y: the sensor dataset, containing the complete set of measurable vehicle behavior measurements, collected simultaneously with X.
- $Y_{r}$: the raw set of measurements of a particular vehicle behavior collected using the sensor. r is the particular vehicle behavior that includes speed, acceleration, steering wheel steering angle, brake pedal angle, accelerator pedal angle, gear angle, and switches angle.
- $Y_{s}$: a more detailed vehicle behavior dataset obtained after processing $Y_{r}$, where s represents more detailed vehicle behavior.
- $X_{i}$: the dataset containing data fields of messages with ID i in X, and $i\in \left(id_{0},id_{1},\ldots ,id_{n}\right)$.
- $Y_{si}$: the result of resampling of $Y_{s}$ according to the frequency of $X_{i}$.
- $R_{si}^{2}$: the coefficient of determination of a multiple linear regression model between $X_{i}$ and $Y_{si}$.
- $\beta_{si}$: the regression coefficient set of the multiple linear regression model between $X_{i}$ and $Y_{si}$.
- $\Delta_{s}$ the threshold value used for the message filter.
- $m_{i}$ the CAN message with ID i.
- $T_{\beta }$: the threshold used for filtering the β.

### 3.1. Data Collection and Processing

**Figure 5:**
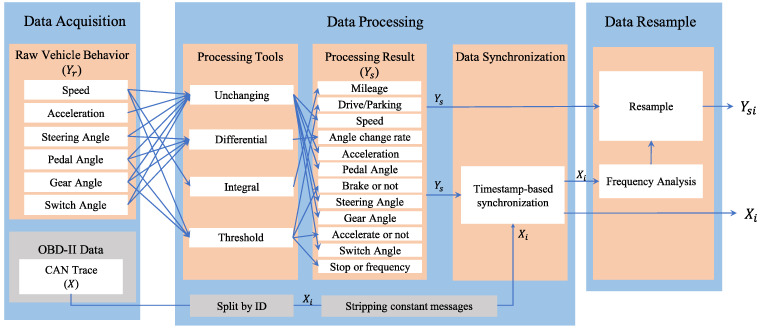
Data collection and processing flow.

This phase aims to acquire and process vehicle behavior measurements, as well as in-vehicle CAN traffic. The flowchart of this phase is shown in Figure 5, which is mainly divided into data acquisition, data processing, and data resampling.

#### 3.1.1. Data Collection

**Figure 6:**
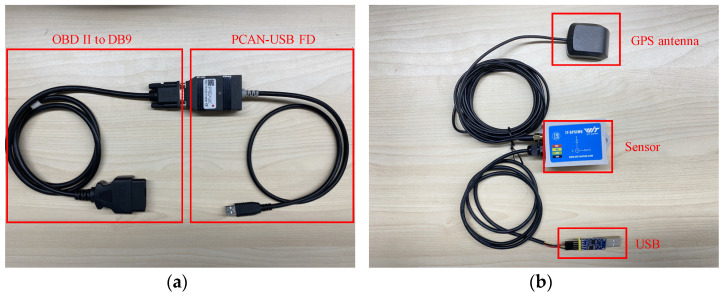
Data acquisition equipment: (**a**) OBD-II data collection equipment; (**b**) Vehicle behavior sensor.

The basic data needed to execute the message reverse framework are the in-vehicle CAN bus traces, X, and the raw physical measurements, $Y_{r}$. Where $Y_{r}$ is the original sensor data for a particular behavior of the vehicle and X is the CAN trajectory obtained when the vehicle performs that behavior. The current phase requires the simultaneous acquisition of X and $Y_{r}$ to reduce errors in linear regression modeling. Therefore, the data acquisition device shown in Figure 6 is used in this phase, using the same timestamp for synchronization. The CAN trace acquisition device is shown in Figure 6a. This device is a combined cable consisting of an OBD-II to DB9 diagnostic cable and a PCAN-USB FD adapter. The cable connects from the OBD-II port of the vehicle to the USB port on the side of the computer to allow the real-time collection of CAN traffic. The behavioral measurements of the vehicle are collected using the sensor device shown in Figure 6b. The device consists of a global positioning system (GPS) antenna, a universal serial bus (USB) interface, and a gyroscope angle sensor with a 0–200 Hz sampling frequency. Although the device is only $78.56 [43], it has a speed sampling accuracy of 0.001 km/h and an angle sampling accuracy of 0.1°. To reduce the error of the sensor sampling, the sampling device should be installed in such a way that the direction of sample change is consistent with the direction of either axis of the sensor. For example, the Y-axis of the sensor is aligned with the head direction when collecting vehicle speed, and the X-axis of the sensor is aligned with the angle change direction when collecting angle data. To represent the behavior and condition of the vehicle as completely as possible, the location of the sensor deployment and the collected data are listed in Table 2. The synchronous work of the above two devices provide the raw data for the reverse framework.

#### 3.1.2. Data Processing and Resampling

Since the raw data collected by the sensors is limited and does not provide a good picture of the various vehicle states, the collected $Y_{r}$ must be processed to reveal more vehicle-related state information. Integral, derivative, and discretization processes are performed on the obtained $Y_{r}$ to get more information. Based on the vehicle behavior in each $Y_{r}$, the rate of behavior change is obtained by derivative, the total amount of change is obtained by integral, and the discrete behavioral states are obtained based on a threshold value. Take speed as an example, the acceleration of the vehicle could be obtained by calculating its derivative to time, and the mileage is obtained by calculating its integral for time. Based on the vehicle speed and the threshold of 1 km/h, the vehicle can be classified into two discrete states of stationary and driving. The data processing methods and results are shown in Table 3. After the extension, there are 13 types of vehicle behaviors. The output after data processing is $Y_{s}$, which contains more detailed vehicle states.

When processing the raw CAN data collected through the OBD-II port, this framework classifies the raw CAN messages based on the ID and removes the constant data field CAN messages. Since the ID identifies the type of the CAN message, $X_{i}$ is first determined by grouping by the ID during processing to facilitate the subsequent modeling of the messages for each ID. Since the framework proposed in this study is based on vehicle behavior to reverse CAN messages, constant CAN messages during sensor acquisition of vehicle behavior do not describe any vehicle behavior and are therefore considered as noise. This noisy data is defined as constant data in READ and LibreCAN, CAN message with constant data fields. Noisy messages can be removed to reduce the number of resamples and subsequent modeling, thus reducing the overall time required.

The next step of data processing is to synchronize the CAN messages with the vehicle behavior. In this study, the CAN messages in $X_{i}$ are selected synchronously with the time interval of the beginning and the end of the vehicle behavior described by $Y_{s}$. Synchronizing the data ensures that the CAN messages in $X_{i}$ and the behavior described by $Y_{s}$ have the same vehicle behavior and state during this time interval.

Finally, multiple linear regression described in Section 2.2 is a method for modeling the dependent and explanatory variables in the same dimension. However, since the messages for each ID appear at a different frequency than the sampling rate of the sensor device, $Y_{s}$, must be resampled based on the frequency of $X_{i}$ to ensure that the two have the same dimensionality [44]. In the data resampling process, this study uses the resampling method of time series in Python to resample each vehicle state $Y_{s}$ according to the frequency of each $X_{i}$ to facilitate subsequent modeling. The resampled data is $Y_{si}$ with the same dimensions as $X_{i}$. In this step, a separate resampling must be performed for each $Y_{s}$ based on the frequency of each $X_{i}$ to obtain $13\times n\;Y_{si}$.

### 3.2. Related Messages Filter

Based on the results of data processing and resampling, the purpose of this stage is to build a linear regression model with $Y_{si}$ as the dependent variable and each bit of the data field in $X_{i}$ as the independent variable. Based on the $R^{2}$ of the model, the messages that are most relevant to the dependent variable are filtered out.

**Figure 7:**
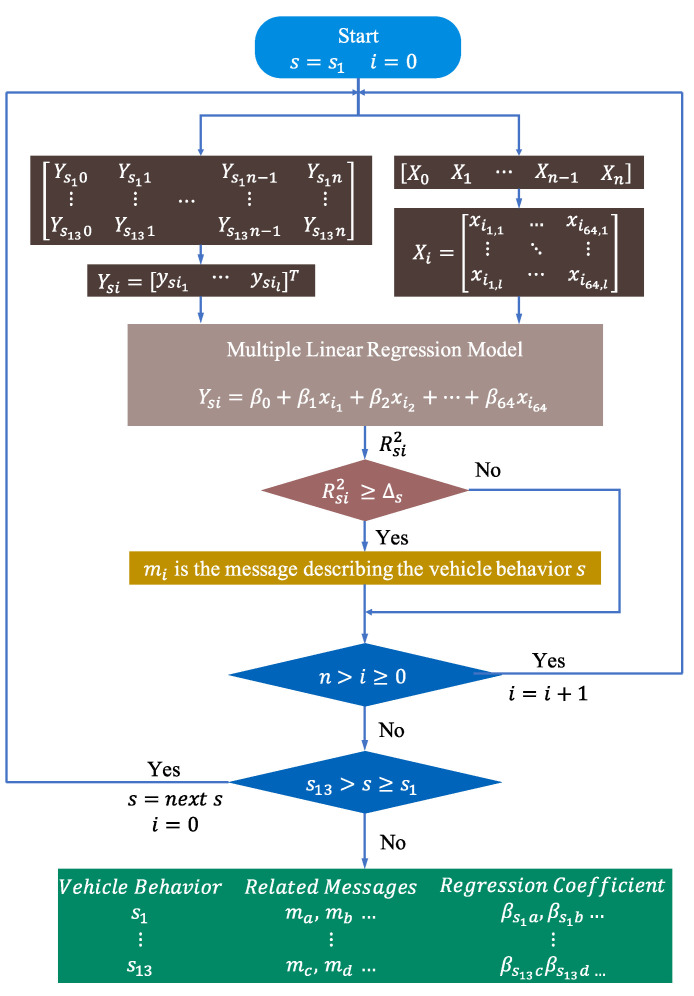
Message selection based on β.

To obtain the relationship between each bit of the data field and the vehicle behavior, this step starts by expanding the data field in $X_{i}$ in bit form, which is an l×64 matrix, where l is the number of messages with ID i. The dependent variable $Y_{si}$, which is an l×1 matrix, is defined to represent the vehicle state data resampled according to the message dimension, where s represents the different vehicle states, $s\in \left(s_{1},\;s_{2},\;\ldots ,s_{13}\right)$. A threshold $\Delta_{s}$ is defined to filter out the best model. The outputs of this stage are messages and linear regression models that are highly correlated with the individual vehicle behavior data. The flow of this phase is shown in Figure 7. The detailed process is shown below.

- **Step 1:** After processing, select a resampled vehicle behavior data $Y_{si}$ and a data set $X_{i}$ with ID i in the CAN bus trajectory.
- **Step 2:** Build a multiple linear regression model with $Y_{si}$ as the dependent variable and $X_{i}$ as the independent variable and calculate the model parameters $R^{2}$ and β.
- **Step 3:** Select the $R^{2}$ obtained in step 2 corresponding to $\Delta_{s}$, and keep only the $R^{2}$ greater than $\Delta_{s}$.
- **Step 4:** Iterate through each $X_{i}$ and repeat step 1 to step 3. According to the filtering result, obtain the most relevant messages and the corresponding models with the vehicle behavior s.
- **Step 5:** Execute step 1 to step 4 for all s to obtain the candidate messages and the corresponding models for each vehicle behavior.

### 3.3. Bit-Level Message Reverse

After the related message filtering phase, the most relevant candidate messages for the particular vehicle behavior and the corresponding linear regression models are determined. The linear regression models of $Y_{si}$ and $X_{i}$ are shown in Equation (7). This result clearly shows the relationship between the vehicle behavior and the data fields of $m_{i}$, where $\beta =\left(\beta_{0},\beta_{1},\ldots ,\beta_{64}\right)$ represents the linear relationship between this vehicle behavior data and each bit of the message.

(7) $$Y_{si}=\beta_{0}+\beta_{1}x_{i1}+\beta_{2}x_{i2}+\ldots +\beta_{64}x_{i64}$$

**Figure 8:**
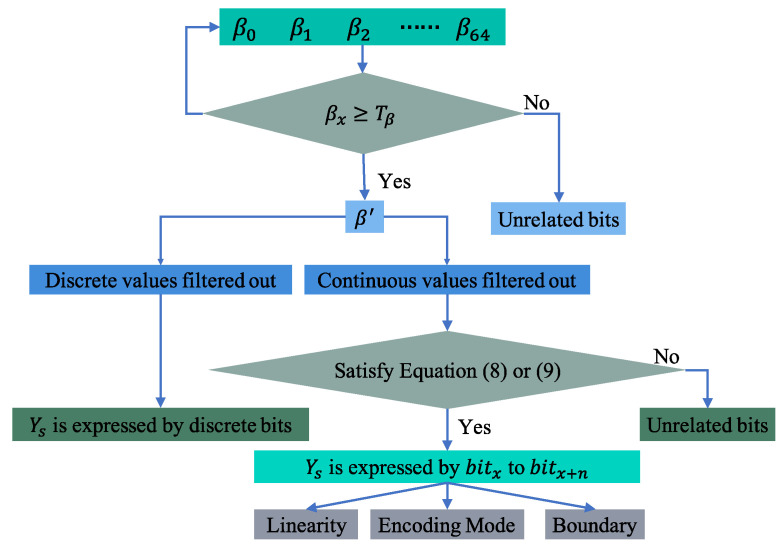
Diagram of bit-level reverse.

In this stage, the specific details of how the data fields of candidate CAN messages describe the behavior of the vehicle are determined by analyzing the regression coefficient β. As shown in Figure 8, the flow of the bit-level reverse for the candidate messages proceeds as follows.

- Iterate through each $\beta_{x}$ in $\beta =\left(\beta_{0},\beta_{1},\ldots ,\beta_{64}\right)$, keeping only those $\beta_{x}$ that are not less than the threshold value. If the value of $\beta_{x}$ is less than the threshold, it means that the xth bit of the data field is not related to the specific vehicle behavior. Otherwise, this bit may represent how the behavior of the vehicle is recorded in the CAN messages. The result after threshold filtering is $\beta^{\prime }$.
- If the filtered $\beta^{\prime }$ is discrete, the corresponding discrete bit likely represents the state of vehicle. If the filtered $\beta^{\prime }$ is continuous, then analyze whether Equation (8) or Equation (9) is satisfied between $\beta^{\prime }$. If satisfied, the bits of the CAN message data field corresponding to the continuous $\beta^{\prime }$ describe the modeled vehicle behavior s. Moreover, the bits satisfying Equation (8) are in Motorola alignment, and those satisfying Equation (9) are in Intel alignment. When not satisfied, the CAN message has no relation to the vehicle’s behavior.
- Analyzing the discrete $\beta^{\prime }$ values and the vehicle state data, the correspondence between the discrete bits and the vehicle state can be obtained reverse. For continuous $\beta^{\prime }$, the data length, the alignment form, and the linear relationship describing the vehicle behavior can be gained.
  (8) $$\beta_{i}=2\times \beta_{i+1}=4\times \beta_{i+2}=\ldots 2^{n}\times \beta_{i+n}$$
  (9) $$\beta_{i}=\frac{1}{2}\times \beta_{i+1}=\frac{1}{4}\times \beta_{i+2}=\ldots \frac{1}{2^{n}}\times \beta_{i+n}$$

## 4. Performance Evaluation

To evaluate the proposed bit-level CAN bus reverse framework, this study implements it on an actual vehicle and obtains specific details of the vehicle CAN message data fields depicting the vehicle behavior for that vehicle. Using the reverse results, the accuracy of the algorithm is evaluated for practical applications based on the available DBC files [45]. In addition, this section evaluates the execution performance of the framework and compares the advantages of the algorithm over other reverse methods. Finally, the advantages of the algorithm in applications are discussed, and an example is given for reversing other vehicle messages when DBC files are not available.

### 4.1. Performance in Real Vehicle

#### 4.1.1. Device Description and Data Processing

**Figure 9:**
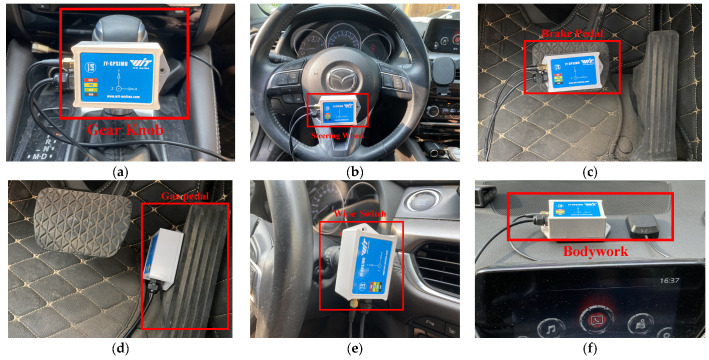
Sensor Acquisition Setup: (**a**) Gear angle; (**b**) Steering wheel angle; (**c**) Brake pedal angle; (**d**) Gas pedal angle; (**e**) Wiper switch angle; (**f**) Vehicle speed.

For the evaluation a 2017 Japanese B-Class sedan was used, whose internal network implements the standard CAN protocol and whose functionality is representative. A DBC file for this model has been obtained, which is used as ground truth for the reverse framework evaluation. To better represent the vehicle behavior, sensors are placed on the body, steering wheel, brake pedal, gas pedal, gear knob, and wiper switch to collect the behavioral data of the vehicle components, which are structured as shown in Figure 9. The CAN data is collected through the OBD-II interface using the combination cable synchronously when collecting vehicle data. The collected CAN data is written to a log file using the upper computer program, containing the ID, type, length, data field, and timestamp of CAN messages. For accuracy evaluation, more than 3,661,000 consecutive CAN bus messages were collected, and more than 5,000,000 vehicle behavior sensor data were sequentially collected in the same period. The dataset (The dataset is partially open source and can be accessed at http://49.232.218.41:8000/data.zip accessed on 23 January 2022) is quantitatively described in Table 4, which describes the measurements and CAN data collected synchronously for each vehicle behavior.

**Figure 10:**
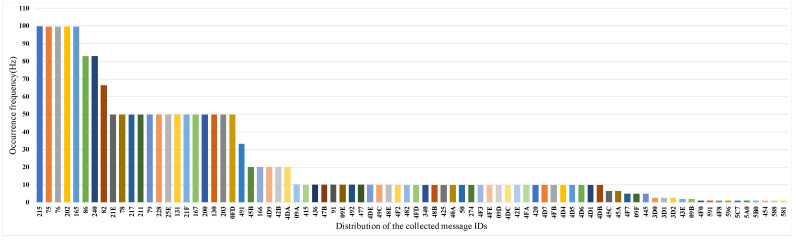
CAN message frequency distribution.

By analyzing the collected CAN traces, the frequency distribution of the messages is shown in Figure 10. This result shows that the number of IDs collected from the test vehicle is 82, which means that there are 82 types of messages in the CAN network. For each type of CAN message, we analyze whether the data field of this CAN message changes and eliminate the messages with unchanged data fields. Based on the analysis and processing of CAN traces, the vehicle behavior data collected in Table 3 is resampled 82 times to obtain $Y_{si}$. A multiple linear regression model is built between $Y_{si}$ and $X_{i}$ according to the message filtering process.

#### 4.1.2. Message Filter Results

**Figure 11:**
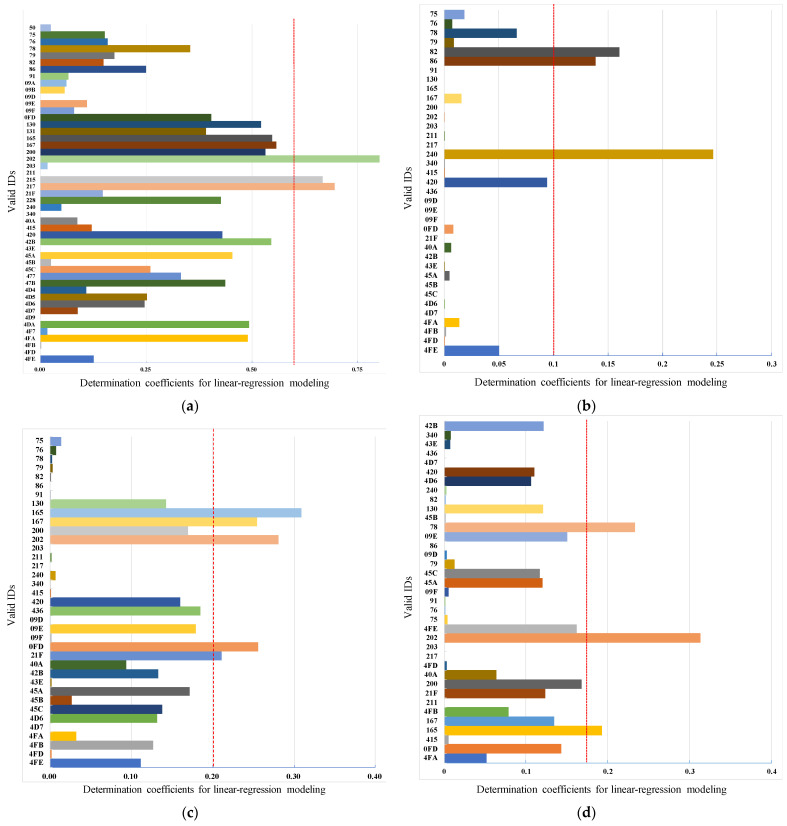

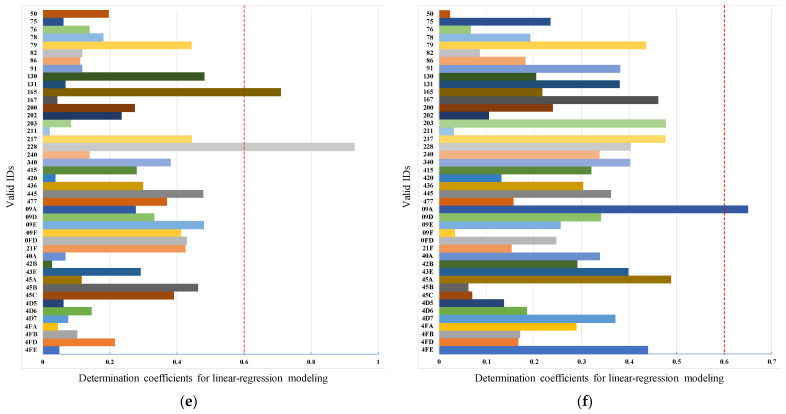
Real vehicle messages filter results: (**a**) speed-related messages; (**b**) steer angle-related messages; (**c**) gas pedal-related messages; (**d**) brake pedal-related messages; (**e**) gear-related messages; (**f**) wiper related-messages.

The results of the multivariate linear regression of the collected continuous vehicle behavior with each type of message are shown in Figure 11. The x-axis is the determination coefficient $R^{2}$ of the multiple linear regression model, and the y-axis is the effective ID distribution.

For the linear regression results of vehicle speed and CAN trace, according to the threshold value 0.6, three types of messages can be filtered out that directly record vehicle speed information with IDs 0x202, 0x215, and 0x217, as shown in Figure 11a. In addition, in this result, there are some $R^{2}$ values close to the threshold, such as 0x130, 0x165, 0x167 and 0x200. This is because they may describe information such as RPM, throttle, etc., that correlate with the vehicle speed, which explains their larger $R^{2}$. However, since these types of vehicle data cannot be collected by sensors, they cannot determine their exact meaning. As shown in Figure 11b, with a $R^{2}$ of 0.1 as the dividing line, the messages IDs strongly correlated with steering wheel angle are 0x086, 0x082, and 0x240. These messages may contain data describing steering wheel torque and steering rate in addition to the information directly representing steering angle. In the same way, messages related to the accelerator pedal are filtered out including messages with IDs 0x165, 0x167, 0x202, 0xFD, and 0x21F, with 0.2 as the divisor, as shown in Figure 11c. Messages with IDs 0x78, 0x202, and 0x165 are categorized as related to brake pedal angle with a threshold of 0.18 as shown in Figure 11d. The results of filtering information related to wiper switch and gear angle are shown in Figure 11e,f. With a threshold of 0.6, the message IDs related to the wiper are 0x9A, and the message IDs related to the gear are 0x165 and 0x228, respectively.

As can be seen from the results of the message filtering, the $R^{2}$ and threshold values for messages related to steering angle, acceleration, and brake pedal are generally small. This result is due to the slight variations in vehicle behavior when collecting these data. For example, the pedal is unlikely to be located at the lowest position when collecting the gas pedal angle while driving. In addition, the results for vehicle speed, gas pedal, and brake pedal show that a certain number of messages have an $R^{2}$ value that is below the threshold, but very close to it. Although these messages do not directly describe the state of the vehicle speed, gas pedal, and brake pedal, they do describe vehicle behavior correlated with the state. For example, the near-threshold telegrams in the throttle results describe the vehicle’s speed, torque, and acceleration, among other things. However, since these messages do not directly describe the vehicle speed, they are classified as irrelevant messages by the threshold. Also, as shown in Figure 11e,f, the $R^{2}$ of the messages related to wiper and gears are clearly distinguished from others. Since the vehicle behavior (gear angle and wiper angle) data and the related CAN messages are all discrete, they can be clearly distinguished from the other messages when the linear regression modeling is performed.

#### 4.1.3. Bit-Level Reverse Results

By analyzing the linear regression result of the filtered messages, it is possible to reverse the portrayal of the vehicle behavior by the individual bits of the message.

**Figure 12:**
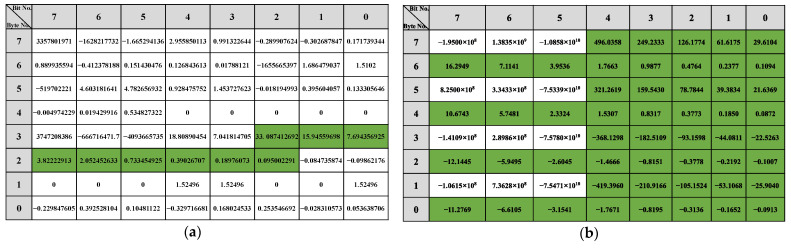

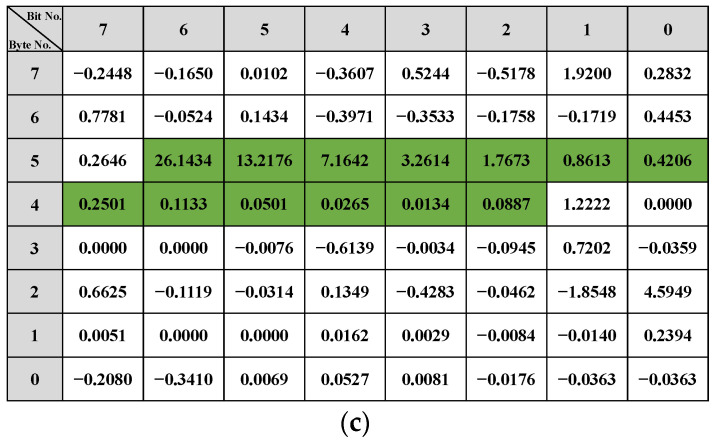
Speed-related messages reverse result: (**a**) ID 0x202 reverse result; (**b**) ID 0x215 reverse result; (**c**) ID 0x217 reverse result.

The reverse result for the speed-related messages is shown in Figure 12. There is a two-fold relationship between the messages with IDs 0x202, 0x215, and 0x217 and the β of the vehicle speed. As shown in Figure 12a, bits 34 to 42 in the message with ID 0x202 indicate the vehicle’s speed, arranged in the format of Motorola. For the message with ID 0x215, according to Figure 12b, bits 0 to 12, bits 16 to 28, bits 32 to 44, and bits 48 to 60 represent the vehicle speed information and the arrangement format is Motorola. The value for the β with ID 0x217 is shown in Figure 12c, and the bits describing the vehicle speed are 34 to 46, and the arrangement format is also Motorola.

**Figure 13:**
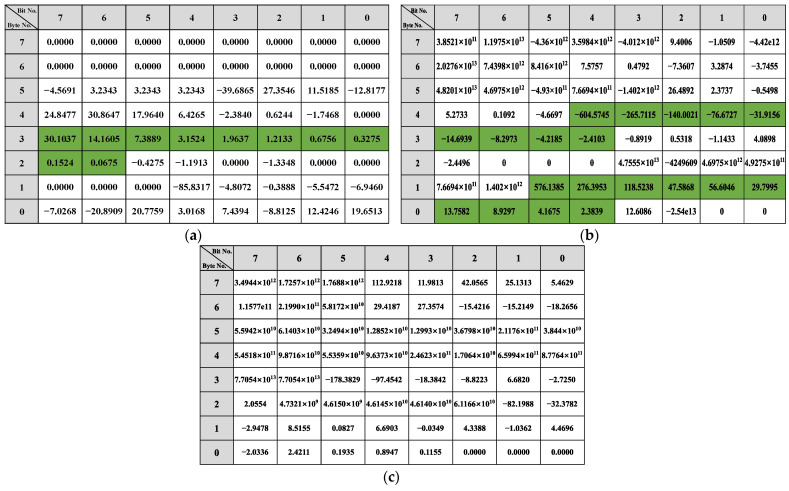
Steer-related messages reverse result: (**a**) ID 0x082 reverse result; (**b**) ID 0x086 reverse result; (**c**) ID 0x240 reverse result.

The reverse results of the steering-related messages are shown in Figure 13. Bits 22 to 31 in the message with ID 0x82 describe the steering angle arranged in Motorola. In the corresponding message with 0x86, the steering angle is specified in bits 3 to 13 and 28 to 36, respectively. The message with ID 0x240 does not describe the steering angle directly, but because its $R^{2}$ is greater than the threshold, it is related to the change in steering.

**Figure 14:**
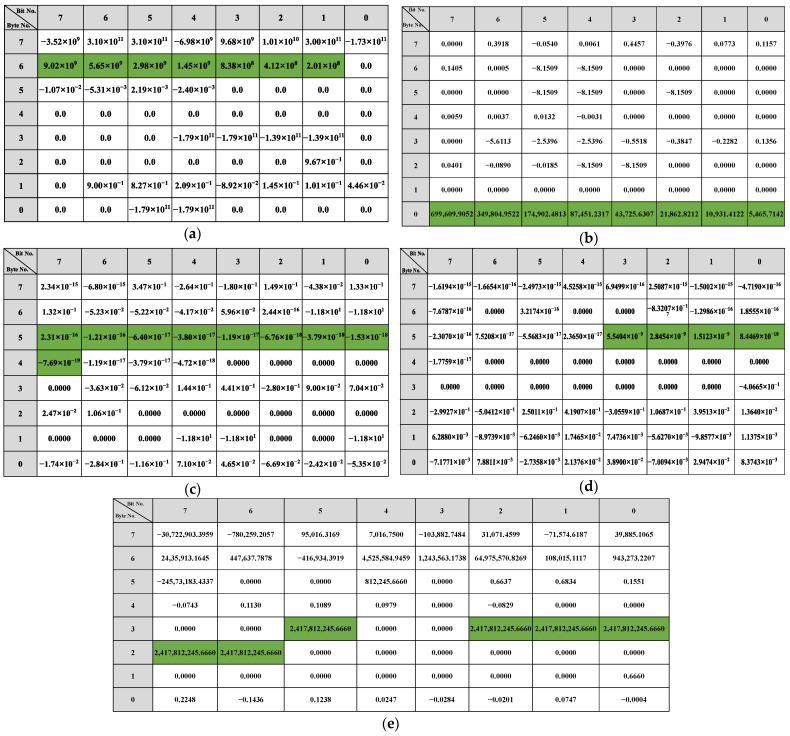
Gas-related messages reverse result: (**a**) ID 0x0FD reverse result; (**b**) ID 0x167 reverse result; (**c**) ID 0x202 reverse result; (**d**) ID 0x21F reverse result with gas angle change rate; (**e**) ID 0x165 reverse result with discrete state.

The results of the throttle-related message are shown in Figure 14. There is an approximate relationship of 2 times in the β corresponding to 0xFD, 0x167, and 0x202 in the results, so based on the β, we find that bits 49 to 55 in the message with 0xFD describe the gas pedal angle. As shown in Figure 14b, in the message whose ID is 0x167, bits 0 to 7 portray the angle of the gas pedal. The angle of the gas pedal in 0x202 is represented in bits 39 to 47. For the messages 0x165 and 0x21F, there is no 2x relationship in β. But the bits 40 to 43 of 0x21F indicate the rate of change of the gas pedal angle as shown in Figure 14d. For 0x165, the gas pedal angle is converted to a discrete state using a threshold: accelerated or not. The result of the discrete value is shown in Figure 14e, from which it can be seen that bit 29, and bits 22 to 26 of ID 0x165 describe whether the gas pedal is activated or not.

**Figure 15:**
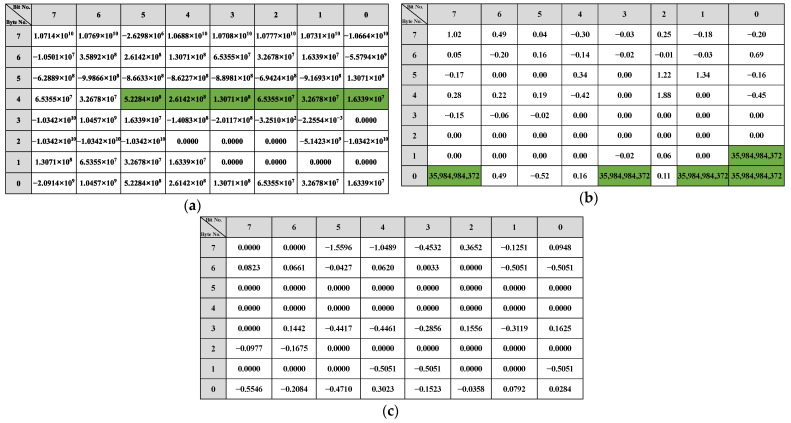
Brake-related messages reverse result: (**a**) ID 0x078 reverse result; (**b**) ID 0x165 reverse result; (**c**) ID 0x202 reverse result.

The results of the bit reverse for the brakes are shown in Figure 15. Based on the β of 0x78, the bits representing the brake pedal are bits 32 to 37, arranged as Motorola. Since there are no significant features in the β of 0x202 and 0x165, the linear regression β of these two types of IDs with discrete states of the brake pedal (braked or not) was calculated using the same method. The results show that in 0x165, bits 0, 1, 3, 7, and 8 indicate whether the vehicle’s state is accelerated or not. For the message with 0x202 as ID, the results show that it does not describe the braking behavior but only the vehicle behavior with respect to braking.

**Figure 16:**
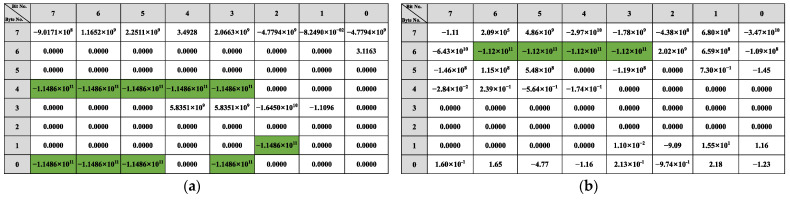
Gear-related messages reverse result: (**a**) ID 0x228 reverse result; (**b**) ID 0x165 reverse result.

**Figure 17:**
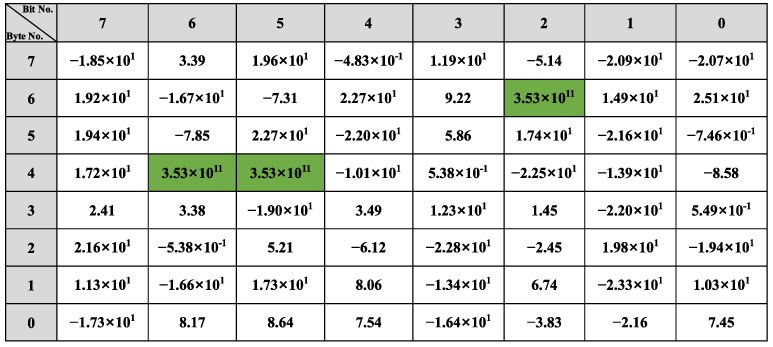
Wiper-related messages reverse result.

The reverse results for the gears are shown in Figure 16. Since the gear behavior data is discrete, it is evident from the β that the message with 0x228 describes the gear information in bits 3, 5 to 7, 10 and 35 to 39, and 0x165 describes the gear in bits 51 to 54. The reverse result of the wipers is shown in Figure 17. The data describing the wiper speed in 0x9A are bits 37 to 38 and bit 50, And the specific reverse results are shown in Table 5.

### 4.2. Framework Accuracy

The accuracy of the system proposed in this study is evaluated using the inverse results of the actual vehicles. The accuracy is evaluated using the DBC files of the test vehicle, which were determined to be the truth.

The accuracy of message filtering is shown in Table 6. All CAN traces are taken from the OBD-II interface, so the accuracy is expressed using the percentage of filtered quantities in the OBD-II. Among all the results, only the brake-related messages have an accuracy of 66.67 %, while all other messages are filtered at 100%. The false-positive result for 0x202 for brakes is due to the fact the brakes are velocity-dependent to some extent. According to the DBC file, 0x202 does contain velocity information, which causes $R^{2}$ to be higher than the threshold. In addition, message 0x240 in the description of the DBC, describes the vehicle’s torque information. Although it is a steering-related message, it cannot be inverted at the bit level because the torque measurement information is not directly available. It is also worth noting that the messages defined in the DBC file do not fully appear in the OBD-II interface. This phenomenon is due to a gateway in the vehicle CAN-bus network, which does not forward all bus traces to OBD-II, but only a portion of the traffic to the OBD-II interface. The rest of the CAN bus data, especially the traffic related to assisted driving and vehicle control, flows only within the vehicle and cannot be captured externally.

**Figure 18:**
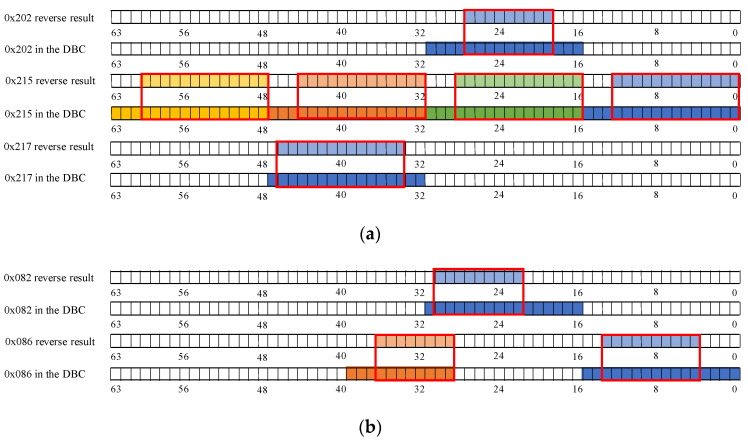

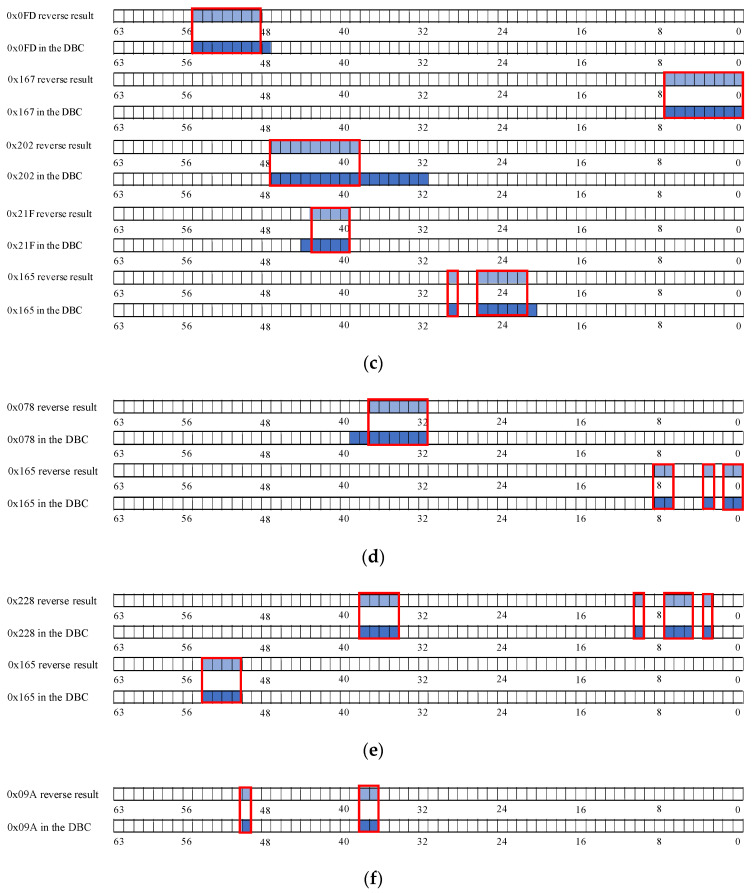
Bit reverse accuracy: (**a**) Speed reverse result; (**b**) Steer reverse result; (**c**) Gas reverse result; (**d**) Brake reverse result; (**e**) Gear reverse result; (**f**) Wiper reverse result.

The bit-reverse accuracy is shown in Figure 18, which compares the bit reverse results of this framework with the vehicle behavior defined in the DBC file. Figure 18a shows the bit-inverse accuracy of the speed-dependent messages. It is observed that bits are written with speed in 0x202, 0x215, and 0x217 are partially reversed to obtain 9 bits for 16 bits in 0x202, 52 bits for 64 bits in 0x215, and 14 bits for 16 bits in 0x217. The bit reversal accuracy of the two steering-related messages, 0x082 and 0x086, is shown in Figure 18b. The proposed framework in this study correctly reverses 9 of the 16 bits in 0x082 and 18 of the 27 bits in 0x086. The accuracy of gas-related message reversal is shown in Figure 18c. 0x0FD gets 7 out of 8 bits, 0x167 completely reverses 8 bits, 0x202 gets 9 out of 16 bits, and both 0x21F and 0x165 have only one bit that is not reversed. Only bits 38 to 39 of 0x078 were not found in the brake-related messages’ reverse results, as shown in Figure 18d. For the gear and wiper-related messages, the bits indicating the gear and wiper switches are both correctly reversed, which can be seen in Figure 18.

The overall bit-reverse accuracy of the proposed framework for vehicle behavior is shown in Table 7. The overall reverse accuracy is over 76%, especially for gear, and wiper reversion can reach 100% because CAN messages and sensor data are discrete and not easily disturbed by other data. The reverse accuracy for vehicle speed, gas pedal, and accelerator pedal are all about 80% because these behaviors are difficult to reach the limit state during vehicle sampling, such as vehicle speed of 255 km/h, gas, and brake pedals kept at the maximum angle. Therefore, when reversing the messages related to these behaviors, their high values can barely be detected (i.e., the high value of β does not satisfy the two-fold relation), which results in poor accuracy. The steering-related information performs the worst, with only 65%. Due to the low degree of steering wheel variability in daily driving, the linear regression model is easily disturbed by irrelevant bits, resulting in poor accuracy of bit reversals.

### 4.3. Time Consumption

The framework’s time performance analysis was performed on a CentOS server with an Intel^®^ Xeon^®^ Gold 6248 CPU @ 2.50 GHz and 8 GB of RAM using Python 3. The time is taken to compute the three critical stages of data resampling, multiple linear regression modeling, and a bitwise inversion was calculated separately during the evaluation. Table 8 shows the execution time results for each phase. The shortest time-consuming stage is the bit-inverse stage, which requires no more than 25 us in the longest case and can be completed within 7 us in the fastest case. The most time-consuming phase is the data resampling phase. The execution time of the data resampling phase varies from 1.15 s to 190.67 s, with an average time of 37.23 s, which is because this stage resamples the sensor data based on the number of IDs that occur. The essential linear regression phase does not take more than 0.84 s. Overall, the time required to reverse the content of a message correctly averages 37.41 s and does not exceed 191.5 s at most.

### 4.4. Result of Comparison with Other Methods

This section presents the performance comparison results between the bit-level reverse framework proposed in this study and other CAN message reverse methods. Nowadays, the effective CAN message reversal algorithms are READ [30], LibreCAN [31], ReCAN, and Bram’s proposed reversal algorithm based on the correlation coefficient [30]. Among them, READ, ReCAN [32], and LibreCAN algorithms use bit-flip rates to delimit CAN message data fields; LibreCAN and Bram’s scheme [37] use correlation coefficients to find the message IDs describing specific vehicle behavior. The differences between the existing algorithms and the linear regression framework in reverse results are given in Table 9. Our proposed scheme is the only one that enables boundary delineation, correlated message identification, and bit reverse. READ and ReCAN only perform CAN message data boundary delineation, Bram’s scheme only addresses correlated message screening, and LibreCAN achieves both results but cannot achieve bit-level inversion. Therefore, this section only compares the performance of this framework with existing algorithms in terms of boundary delineation, correlated message filtering, and execution complexity.

#### 4.4.1. Boundary Delineation

In terms of boundary delineation, we compare the linear regression framework of this paper with the bit-flip rate algorithm used by READ, ReCAN, and LibreCAN. The performance of the methods in this study and the bit-flip rate method in delineating CAN messages with discrete states and continuous vehicle behavior is shown in Table 10. The framework in this paper can delineate the vehicle behavior within the corresponding range with 100% correctness, while the bit-flip-based rate is only 53.3% correct in delineating the boundaries. In particular, bit flipping has relatively good results in delineating CAN messages describing continuous behavior, but boundary delineation errors occur for fields corresponding to discrete vehicle behavior.

**Figure 19:**
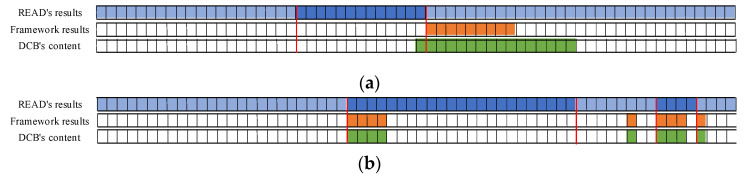
Boundary division results of bit-flip rate and proposed method: (**a**) Continuous value division result (0x082 for steering); (**b**) Discrete value division result (0x228 for gear).

The reasons for the different performance of existing methods in delineating boundaries are explained in Figure 19 using 0x082 (for steering) and 0x228 (for gears) as examples. As shown in Figure 19a, this approach may not set the boundary for the boundary delineation of continuous values quite correctly, but the delineation is within the correct range. In contrast, the bit-flip rate approach is easily affected by bits with the exact change pattern or are completely changed when dividing the boundary, which leads to the boundary division outside the normal range. Figure 19b compares the delineation results of the two methods for discrete values. The bit-flip rate approach fails to delineate the boundary accurately because the flipped cases of individual bits are generalized to the same field as the adjacent invariant bits when delineating the boundary. Therefore, the framework proposed in this study gives better results for discrete values.

#### 4.4.2. Related Message Filtering

**Figure 20:**
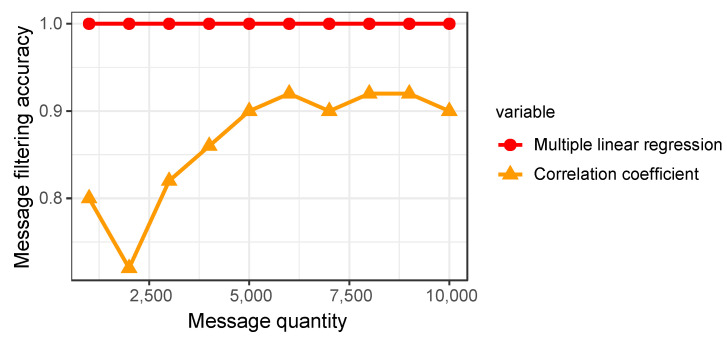
Comparison between correlation coefficient and multiple linear regression.

This section describes the outstanding performance of the framework in this paper compared to existing schemes in related message filtering, where existing schemes mainly use correlation coefficients (e.g., LibreCAN, Bram’s method) to filter related messages. Figure 20 compares the performance between our proposed framework and the Pearson correlation coefficient for correlated message filtering. Regardless of the number of messages, the multiple linear regression method proposed in this study can filter messages related to vehicle behavior with 100% accuracy. When using the correlation coefficient to filter messages, although the accuracy of candidate message filtering increases as the number of messages rises, the accuracy still does not exceed 95%. When calculating the correlation between the two vectors, the results of the Pearson correlation coefficient are easily influenced by outliers in the two vectors, resulting in a reduced correlation coefficient that does not effectively filter out candidate messages [46]. In this paper, using multiple linear regression to model each bit of the data field as an independent variable, the effect of outliers is weakened, and the relevant messages are effectively filtered out. This result shows that the framework proposed in this study is more accurate than existing message filtering methods.

In addition, as shown in Table 11, the accuracy of the linear regression method is not affected by the number of messages, which remains 100%, while the correlation coefficient requires a higher number of messages to obtain a higher correct rate. This indicates that fewer messages are needed to locate messages related to vehicle behavior when using the linear regression method for CAN message screening, reducing data acquisition and computation time that speeds up the reverse work.

#### 4.4.3. Execution Complexity

We compare the algorithms in this section concerning the devices needed for their execution, the data requirement, the algorithm execution time, and the reverse results. As shown in Table 12, each algorithm relies on OBD-II data acquisition devices. Only the framework and LibreCAN require additional sensor devices and smartphones, respectively. In terms of data requirements, the READ and ReCAN require only CAN traffic, the linear regression method and LibreCAN require data from additional devices. However, the correlation coefficient method requires UDS data through interaction with the vehicle [47]. LibreCAN is the algorithm that takes the longest time to execute since some manual work is also required, and the fastest execution is the correlation coefficient method. The framework in this paper is close to the average time of the READ algorithm. However, in terms of reverse results, our scheme is the only one that can achieve bit-level reverse, outperforms the other algorithms in boundary delineation and message filtering, and does not require interaction with the vehicle. Although additional sensor devices are required, such sensors can be purchased very cheaply and used very simply in the market.

### 4.5. Application and Discussion

#### 4.5.1. Application

The bit-level automotive CAN bus reverse framework proposed in this study can be used in almost all commercially available vehicles, independent of vehicle make and model. According to Table 12, the implementation of the framework requires OBD-II [48,49] data collection devices, sensors, and CAN traffic. In-vehicle CAN network traffic is typically collected using the OBD-II interface, a globally accepted automotive standard. It is required for almost all commercially available vehicles to be equipped with an OBD-II interface before they can be marketed [50,51,52,53,54]. Therefore, regardless of vehicle models on the market, the vehicle CAN traffic can be obtained after connecting OBD-II data collection devices. Therefore, regardless of vehicle models on the market, the vehicle CAN traffic can be obtained after connecting OBD-II data collection devices. For OBD-II data acquisition devices, such devices are readily available on the market today, with prices ranging from a few tens to a few hundred dollars. The sensor devices used in this framework are off-the-shelf motion sensors, which are inexpensive and easily placed in various vehicle parts to collect relevant data. Using CAN traffic and sensor data as input to our proposed framework, the algorithm proposed in this paper can obtain how CAN messages in any vehicle describe the vehicle state.

To verify the applicability of the framework, an electric car with completely a different power and brand was chosen to apply the framework. The reverse results are shown in Table A1 in Appendix A. In the absence of relevant DBC files, a script is provided in the appendix that can display CAN data changes in real-time to confirm the accuracy of each result. All filtered messages are consistent with the actual results in the actual results, and the reverse results of the bits remain consistent with the data bit changes. Overall, the method proposed in this study can be applied to most vehicle CAN message inversions and is not affected by vehicle changes.

#### 4.5.2. Discussion

In this study, we propose an innovative bit-level reverse framework for automotive CAN messages. This framework builds a multiple linear regression model between CAN traces and sensor data, uses decision coefficients to filter candidate messages, and uses model parameters to determine how data fields represent vehicle behavior and maximally recover DBC files. In the test vehicle, this framework has high accuracy in both message screening and bit-inversion. However, the limitation of the test environment results in the unavailability of the extreme vehicle behavior data, leading to less than perfect results in bit-reversion. In addition, the framework reverses the candidate messages correctly in a short time, which improves the reversal efficiency. Our study proposes the only CAN message translator that can achieve bit-level reversal and has significant advantages over other existing methods for boundary delineation and message verification. Finally, the framework can be applied to any standard-compliant commercially available vehicle.

## 5. Conclusions

### 5.1. Implication

This study examines the bit-level CAN bus reverse framework using a multiple linear regression model. This framework is the only method that can achieve bit-level reversion. It uses sensor data as the dependent variable and each bit of the CAN message data field as the dependent variable to build a multiple linear regression model to obtain the carving of vehicle behavior for each bit based on the β. This study shows that the framework can accurately filter CAN messages related to vehicle behavior, reverse the way each bit represents vehicle behavior, and obtain the length, boundary, and alignment format of the signal. Compared to other methods, the framework can delineate the signal length and message filtering more accurately. In addition, the algorithm uses a globally available standard interface (OBD-II) and common motion sensors to capture CAN traffic and vehicle behavior data, which allows access to data that is not limited by model and make, making the algorithm more usable. The excellent reverse capability of the system can help automotive security researchers to quickly discover how CAN messages describe vehicle behavior when DBC files are not available. It is worth mentioning that attackers may also use our approach to find better attack approaches against cars. Although the framework makes DBC files less secret, it is more meaningful to study the automotive CAN detection and defense attack capabilities. In addition, a better attack prevention system could be developed based on the reverse results of this scheme.

### 5.2. Limitations and Future Work

The present study has three significant limitations that can be addressed in future studies.

First, the lack of extreme data affected the correctness of the experiment. When CAN traffic and vehicle behavior data were acquired, CAN data and sensor data could not cover extreme data, such as vehicle speed reaching 255 km/h, maximum steering wheel angle, and pedal reaching maximum angle. The lack of extreme data departs the highest position in the experimental results, resulting in unsatisfactory experimental results. Future research can obtain extreme data in closed scenarios to optimize the experimental results.

Second, insufficient DBC files. We use open-source DBC descriptions as truth when testing the results of validation experiments in vehicles. However, most of the current open-source DBC files are obtained by extracting the ECU firmware, resulting in a minimal number. This study can obtain the description of CAN messages without firmware, which provides a new idea to obtain DBC files for subsequent studies.

Finally, application limitations. Due to the limited number of test vehicles used, this framework validated its reverse effect in a subset of vehicles. According to the devices and data on which the framework relies, it can be applied to almost all vehicles. To address the difficulty of testing in actual vehicles, software and hardware simulations [55] of the internal networks of vehicles can be investigated in future research to address the application limitations. 

## Figures and Tables

**Table 1:** DBC file content definition

Field Name	Definition
Name	The overall function of this message (e.g., body, speed, etc.)
ID	The identifier of this message
Cycle time	The sending period of this message
Length	The length of this message
Function	The specific function contained in this message (e.g., angel change)
Byte order	The arrangement of the specific function
Start byte	The starting byte of the specific function
Start bit	The starting bit in first byte
Bit length	The length of the function
Unit	The unit of the function
Resolution	The resolution of the function
Offset	The offset of the function

**Table 2:** Sensor locations and associated physical value.

Location	Physical Characteristics
Bodywork	Speed, Acceleration
Steering wheel	Steering angle
Brake pedal	Pedal angle
Accelerator pedal	Pedal angle
Gear knob	Gear angle
Wiper switch	Switch angle

**Table 3:** Methods and results of raw data processing.

Raw Data (*r*)	Operation	Detailed Vehicle Behavior (*s*)
**Speed**	-	Speed
	Integrals	Mileage
	Judgment by threshold	Drive/Parking
**Brake Pedal Angle**	-	Brake pedal angle
	Differential	Angle change rate
	Judgment by threshold	Brake or not
**Accelerator Pedal Angle**	-	Accelerator pedal angle
	Differential	Angle change rate
	Judgment by threshold	Accelerate or not
**Gear Angle**	-	Gear angle
	Judgment by threshold	P/R/N/D
**Wiper Switch Angle**	-	Wiper switch angle
	Judgment by threshold	Stop or frequency

**Table 4:** Number of vehicle behaviors and CAN messages.

Vehicle Behavior	Number of Sensor Record	Number of CAN Messages
Bodywork	298,649	1,769,768
Steering Wheel	16,148	132,122
Brake Pedal	7961	57,399
Accelerator Pedal	6364	60,772
Gear Handle	13,105	113,001
Wiper Switch	12,876	118,095

**Table 5:** Results for gears and wipers of bit-level reverse.

Gear	**Status**	**ID 0x165**	**ID 0x228**			
		**Bits 54–51**	**Bits 39–35**	**Bit 10**	**Bits 7–5**	**Bit 3**
	P/N	0110	00010	1	110	0
	D	1100	10000	1	001	1
	R	1101	00010	1	010	1
Wiper	**Status**	**ID 0x09A**				
		**Bit 50**		**Bits 38–37**		
	Auto	1		10		
	Slow	0		10		
	Fast	0		01		

**Table 6:** Message filtering accuracy results for vehicle behavior.

Behavior	DBC Defined Messages	Messages Captured from OBD-II	Framework Filtering Results	Accuracy
Speed	0x25E, 0x217, 0x202, 0x215, 0x35F, 0x361	0x217, 0x202, 0x215	0x217, 0x202, 0x215	100%
Steer	0x86, 0x240, 0x243, 0x82	0x86, 0x240, 0x82	0x86, 0x240, 0x82	100%
Gas	0x202, 0x21C, 0xFD, 0x167, 0x165, 0x21F	0x202, 0xFD, 0x167, 0x165, 0x21F	0x202, 0xFD, 0x167, 0x165, 0x21F	100%
Brake	0x165, 0x78	0x165, 0x78	0x165, 0x78, 0x165	66.67%
Gear	0x228, 0x165	0x228, 0x165	0x228, 0x165	100%
Wiper	0x9A	0x9A	0x9A	100%

**Table 7:** Bit reverse result with DBC file description.

Vehicle Behavior	Number of Relevant Bits in DBC	Reverse Results	Accuracy
Speed	96	74	77.1%
Steer	43	28	65.1%
Throttle	44	34	77.3%
Brake	13	11	84.6%
Gear	13	13	100%
Wiper	3	3	100%
Total	212	163	76.9%

**Table 8:** Implementation time of each stage.

Step	Shortest (s)	Longest (s)	Average (s)
Resample	1.150728	190.674251	37.23192305
Linear regression model	0.007088	0.83345	0.179022554
Bit reverse	0.000007	0.000025	0.0000099
Total	1.157823	191.50772	37.4109555

**Table 9:** Reverse function compared with existing algorithms.

Algorithm	Boundary Delineation	Related Message Filtering	Bit-Level Reverse
Bit-level reverse based on linear regression	√	√	√
READ	√	×	×
LibreCAN	√	√	×
ReCAN	√	×	×
Reverse engineering based on correlation coefficient	×	√	×

**Table 10:** Boundary Delineation Comparison.

Vehicle Behavior	ID	Linear Regression	Bit Flip (READ, ReCAN, LbreCAN)
Speed	202	√	√
	215	√	√
	271	√	√
Steer	082	√	√
	086	√	×
Throttle	0FD	√	×
	167	√	×
	202	√	√
	21F	√	√
	165	√	×
Brake	078	√	√
	165	√	√
Gear	228	√	×
	165	√	×
Wiper	09A	√	×
Total Accuracy		100%	53.33%

**Table 11:** The influence of different message counts on accuracy.

Methods	Number of Messages									
	1000	2000	3000	4000	5000	6000	7000	8000	9000	10,000
Linear regression	100%	100%	100%	100%	100%	100%	100%	100%	100%	100%
Correlation coefficients	80%	72%	82%	86%	90%	92%	90%	92%	92%	90%

**Table 12:** Execution complexity comparison of different algorithms.

Algorithm	Devices Requirements	Data Requirements	Average Time	Reverse Results
Bit-level reverse based on linear regression	OBD-II data acquisition device, Behavior sensors	CAN traffic, Sensors data	37 s	Boundary Delineation, Related message filtering, Bit-level reverse
READ	OBD-II data acquisition device	CAN traffic	35.9 s	Boundary Delineation
ReCAN	OBD-II data acquisition device	CAN traffic	35.9 s	Boundary Delineation
LibreCAN	OBD-II data acquisition device, Smartphone	CAN traffic, Smartphone data	>60 s	Boundary Delineation, Related message filtering
Reverse engineering based on correlation coefficient	OBD-II data acquisition device	CAN traffic, UDS data	<20 s	Related message filtering

## Data Availability

The data presented in this study are available in Section 4.4.1.

## Appendix A

Table A1 shows the framework’s CAN message reverse for an electric vehicle manufactured in China. Although there is no DBC file to verify its correctness, we wrote a script (It can be found at http://49.232.218.41:8000/ accessed on 23 January 2022) that can display the data changes of the specified ID in real-time using the experimental equipment in this paper to verify the correctness of the results.

**Table A1:** Another vehicle reverse result.

Behavior	ID	Bits	Description
speed	0x212	48–56	real-time speed data
	0x23A	32–40, 56–64	real-time speed data
	0x21A	17–32	real-time speed data
mileage	0x21A	48–64	mileage per unit of time
steer	0x236	58–64	real-time steering data
brake pedal	0x668	0–16	brake pedal angle
	0x668	36	brake status
accelerate pedal	0x668	17–31	accelerate pedal angle
gear	0x235	39, 42, 44	D
		39, 42, 43	R
